# Non-Invasive Device for Blood Pressure Wave Acquisition by Means of Mechanical Transducer

**DOI:** 10.3390/s19194311

**Published:** 2019-10-05

**Authors:** David Zambrana-Vinaroz, Jose Maria Vicente-Samper, Carlos G. Juan, Vicente Esteve-Sala, Jose Maria Sabater-Navarro

**Affiliations:** 1 Neuroengineering research group, Miguel Hernández University of Elche, 03202 Elche, Spain; jose.vicentes@umh.es (J.M.V.-S.); carlos.juan01@umh.es (C.G.J.); 2 Department of Software and Computing Systems, University of Alicante, 03690 Alicante, Spain; vesteve@ua.es

**Keywords:** blood pressure, carotid, dicrotic notch, dome, ECG, HRV, mechanical transducer, pressure sensor, PTT

## Abstract

Blood pressure wave monitoring provides interesting information about the patient’s cardiovascular function. For this reason, this article proposes a non-invasive device capable of capturing the vibrations (pressure waves) produced by the carotid artery by means of a pressure sensor encapsulated in a closed dome filled with air. When the device is placed onto the outer skin of the carotid area, the vibrations of the artery will exert a deformation in the dome, which, in turn, will lead to a pressure increase in its inner air. Then, the sensor inside the dome captures this pressure increase. By combining the blood pressure wave obtained with this device together with the ECG signal, it is possible to help the screening of the cardiovascular system, obtaining parameters such as heart rate variability (HRV) and pulse transit time (PTT). The results show how the pressure wave has been successfully obtained in the carotid artery area, discerning the characteristic points of this signal. The features of this device compare well with previous works by other authors. The main advantages of the proposed device are the reduced size, the cuffless condition, and the potential to be a continuous ambulatory device. These features could be exploited in ambulatory tests.

## 1. Introduction

Blood pressure is a very important parameter for the screening of the circulatory system. As a matter of fact, it is one of the major cardiovascular complication factors, as the World Health Organization (WHO) pointed out that high blood pressure is the most common cause related to preventable death in developed countries [1]. It affects about one in four adults and provokes a life expectancy reduction of approximately 10–15 years [2]. Currently, the sphygmomanometer is used to measure blood pressure. It is a medical instrument used for indirect blood pressure measurement and can provide the measurement in millimeters of mercury (mmHg). To carry out the measurement, it is necessary to have an inflatable cuff system and a manometer [3]. However, in the clinical setting, when the patient is unstable and under vasoactive treatments, it is indicated to get access to the artery through a cannula and measure the blood pressure directly, as this allows to obtain data continuously and more precisely than using an indirect measurement (sphygmomanometer) [4]. Some of the methods and systems available in the market for directly analyzing the pulse wave are the following: PiCCO® (Pulsion) [5], PulseCO® (LiDCO) [6], or MostCare® (Vygon) [7].

On the one hand, the direct measurement is invasive, needing to cannulate an artery to provide for intraarterial pressure measurement. On the other, the indirect measurement is uncomfortable owing to the bracelet being inflated and oppressing the patient, and it is not continuous. For these reasons, new techniques arise to carry out the continuous measurement of blood pressure in a more comfortable way [8]. Within these new techniques, a promising one is the use of photoplethysmography (PPG) signal to get the pulse transit time (PTT) [9].

It should be noted that PPG is an optical technique used to detect volumetric changes in the blood in the peripheral circulation. It is also used to analyze the blood pressure waveform. This technique relies on the principle that blood absorbs more infrared light than other tissues. The resulting signal contains important information about heart rate variability (HRV) or blood pressure, among others. These signals are of great importance for clinical applications such as physiological biomarkers monitoring, vascular assessment, or autonomic function analysis [10]. Many current devices use the PPG signal to carry out the calculation of the PTT signal [11,12].

PTT is the time interval of the propagation of the pulse wave between two arterial sites [13,14] and it gives an inversely proportional estimation of the blood pressure. This way, high blood pressure corresponds to high pulse wave velocity (PWV), which in turn implies low PTT value [15,16], as can be seen in Equation (1), with *l* being the vessel length. Thanks to the Moens–Korteweg equation, it is possible to establish the relationship between pulse wave velocity (PWV) and the modulus of elasticity of the artery *E*, the thickness of the arterial wall *h*, the diameter of the artery *d*, and the blood density *ρ* (Equation (1)). In the work of [17], a monitoring device based on the transit time of the pulse without bracelet (PTT) is proposed for ambulatory monitoring of blood pressure. For this purpose, the correlation of PTT and blood pressure was evaluated.

(1) $$\mathrm{PWV}=\frac{\Delta x}{\Delta t}=\frac{l}{\mathrm{PTT}}=\sqrt{\frac{Eh}{\rho d}}.$$

A recognized family of systems used clinically for the analysis of the blood pressure waveform is SphygmoCor [18]. These devices use different techniques to perform blood pressure wave measurements (occlusive cuff, tonometry). SphygmoCor technology can obtain the pulse wave from the carotid and femoral arteries and estimates PWV from the delay between pulse waves on the carotid and femoral arteries with respect to electrocardiogram (ECG) [19,20]. In addition, over the past two decades, HRV has been extensively investigated and represents one of the most promising markers for cardiac autonomic nerve function. The heart rate variability is measured as the variation in the time interval from beat to beat [21].

The device presented in this article is part of a more complex system that monitors biomedical variables (ECG, accelerometer, galvanic skin response (GSR)) on an outpatient basis in patients suffering from epileptic seizures. Therefore, the device described aims to monitor cardiovascular variables such as ECG, PTT, and HRV. This article shows a device capable of recording the blood pressure wave in a non-invasive way, which can be used for ambulatory applications, using a mechanical transducer. In addition, it allows to calculate other cardiac system monitoring parameters such as HRV and PTT by combining this wave with the acquisition of the ECG signal. Next, in the materials and methods section, we will describe the characteristics of the proposed device. The materials used will be also shown, as well as the device operation and the process of acquisition of the pressure wave data. The results show how the pressure wave has been successfully obtained in the carotid artery area, discerning the characteristic points of this signal. In addition, the improvements of this device compared with other similar ones will be discussed.

## 2. Materials and Methods

### 2.1. Device Description and Implementation

The device presented in this article is capable of capturing the vibrations (pressure waves) produced by the carotid artery in a non-invasive manner by means of a pressure sensor encapsulated in a closed dome filled with air and without using an occlusive cuff. By placing the device onto the outer skin of the area of the carotid artery, the vibrations due to the blood pressure changes will exert a deformation in the dome. An increase in the inner pressure in the dome will consequently be given, which will be measured by the pressure sensor inside the dome. Thanks to these vibrations, it is possible to obtain an adequate resolution of the characteristic points of this wave comparable to the ones obtained with invasive devices. 

**Figure 1:**
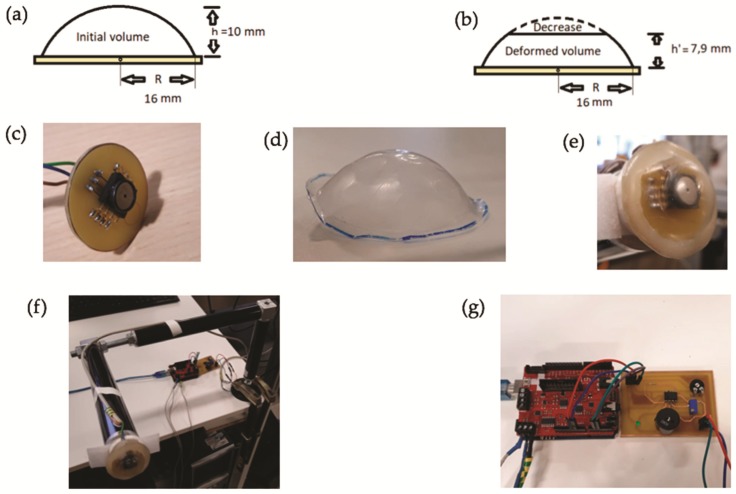
(**a**) Dome in the initial position without deformations; (**b**) deformed dome when it is placed in the area of the carotid artery; (**c**) printed circuit board (PCB) designed in order to acquire the pressure signal with the analog sensor MPXA6115A6U; (**d**) dome; (**e**) the dome glued onto the PCB; (**f**) fixed support system anchored to the table; (**g**) buzzer warning system.

The ratio of volumes and pressures between the dome in the initial position (without deformations) and the deformed dome can be obtained when it is placed in the area of the carotid artery exerting a constant force (Figure 1a,b). As the dome is a hemispherical cap, it is possible to obtain the initial volume of the dome (3.977 cm^3^). The initial pressure inside the dome is 103.25 kPa. Knowing the deformation that occurs when the device is placed on the carotid area, as well as its resulting dimensions (shown in Figure 1b), the new volume of the dome can also be calculated (roughly 3.767 cm^3^ in the case shown). Later, the pressure reaching the air inside the dome is 109.05 kPa, obtained with the sensor inside the dome. That yields a 5.62% pressure change when the volume changes by 5.57%. Therefore, the pressure shows relative changes comparable to those from the volume, thus providing for a suitable transduction.

The selection of a suitable sensor is crucial for this device, regarding the reduced space available. At the beginning, a digital pressure sensor was used, specifically the BMP280 model from Bosch. The main reasons for choosing this model were the small size of the sensor (2.5 × 2.5 mm^2^) and the ability to continuously record small pressure changes. In addition, it had the advantage of incorporating different communication protocols (I2C and SPI) that made it very easy to perform the reading of pressure values using a microcontroller. Despite all the ideal characteristics described, the maximum sampling frequency was very low (less than 30 Hz) for this application and there was not enough resolution to discern the different key points in a blood pressure wave.

We thus decided to choose an analog sensor (Figure 1c), specifically the model MPXA6115A6U from NXP, which provides a voltage signal as a function of the measured pressure. It has a sensitivity of 45 mV/kPa. The main disadvantage of this sensor is its size (10.5 × 18 mm^2^). With this sensor, an adequate resolution was obtained to discern the different key points in a blood pressure wave. Figure 1c shows the printed circuit board (PCB) that was designed to be able to acquire the pressure signal with the analog sensor.

The Arduino Uno microcontroller was used to read the voltage values provided by the analog sensor, related to the measured pressure values. One of the analog pins was used for such a purpose, setting the sampling frequency of the analog sensor to 2 ms (∼500 Hz). The voltage values were transformed to pressure values according to the sensor characteristics, and these pressure values were then stored for further treatment and analysis. The dome acts as a mechanical transducer, capturing the vibrations of the carotid artery. These vibrations increase the air pressure inside the dome and the pressure sensor is able to record the changes that occur. A 0.125 mm thick polypropylene sheet was used to design the dome. To give it the shape of a dome, first, a thin board was designed with a hole of a diameter slightly smaller (28 mm of diameter) than the size of the PCB (32 mm of diameter). In addition, a hemispherical piece was also designed with the desired shape of the dome. Then, with the sheet of polypropylene stretched over the hole in the board, a gentle pressure was exerted with the hemispherical piece, which was previously heated, pushing the polypropylene sheet through the hole to deform it and give it the desired dome shape (Figure 1d).

Later, the dome was glued onto the PCB perimeter by means of an epoxy resin consisting of epichlorohydrin and bisphenol A (Figure 1e). Owing to the small dimensions of the dome and the PCB, the epoxy was painstakingly applied with a needle to both the PCB and the dome, and then both pieces were stuck. Thanks to the shape of the dome, when it is finally sealed, the air remains inside. It should be noted that, to ensure the device was working properly, it was important to seal very well the joint between the dome and the PCB in order to prevent leaks. If there was any leakage, the pressure inside the dome would not increase suitably according to the carotid artery vibrations. The sensitivity of the mechanical transducer is approximately 38 mmHg/N.

With the dome being properly sealed, because the pressure values are obtained inside it, the pressure values will increase as a result of exerting a hand force when placing or holding the device to make measurements. Because of this reason, a fixed support system was anchored to a table, being the non-invasive device attached at the end of the support (Figure 1f). This way, the above-mentioned problem was avoided when coupling the device to the user’s neck area, where the carotid artery is located. With this support, the user can move so that the device is finally placed in the right position in a comfortable way, avoiding the pressure fluctuations when holding it with the hand. It should be noted that the quality of the recorded signal will strongly depend on the proper placement of the device, as expected in these techniques [18,19,20]. The stronger the force exerted against the sensor, the higher the quality of the blood pressure wave. However, it is uncomfortable to feel the force of the sensor in the area of the carotid artery. Therefore, after a multitude of tests, it could be seen that when a pressure between 108 and 109 kPa was reached inside the dome, the exerted force was still comfortable, and the resolution of the wave was close to the optimal one. A buzzer warning system was implemented (Figure 1g), which warns when the pressure values recorded by the sensor are within a very limited range of 108 kPa to 109 kPa (810.06–817.56 mmHg). This ensures that the force exerted by the user against the device at the end of the support remains constant and is optimal. Therefore, when the measurements are being made correctly, a buzzer beep sounds.

Furthermore, the ECG signal is also acquired in the designed system through an Arduino module called e-Health [22]. The e-Health module is a system compatible with Arduino boards and is capable of acquiring different signals such as pulse, oxygen in blood (SpO2), airflow (breathing), body temperature, electrocardiogram (ECG), glucometer, galvanic skin response (GSR—sweating), blood pressure (sphygmomanometer), patient position (accelerometer), and muscle/electromyography (EMG). The ECG signal is recorded by means of three electrodes. This module makes use of all the digital pins of Arduino; therefore, it was necessary to use an analog pin in order to control the buzzing system. To do so, an astable circuit was used thanks to the integrated circuit 555 (integrated circuit commonly used in the generation of timers, pulses, and oscillations) to generate an oscillatory wave that makes the buzzer sound. In addition, a BJT transistor (BD139) was used in saturation configuration to switch on and off the power supply for the circuit, as the analog output of the Arduino was not capable of reaching 5 volts, the minimum voltage needed for the proper functioning of the designed circuit. 

**Figure 2:**
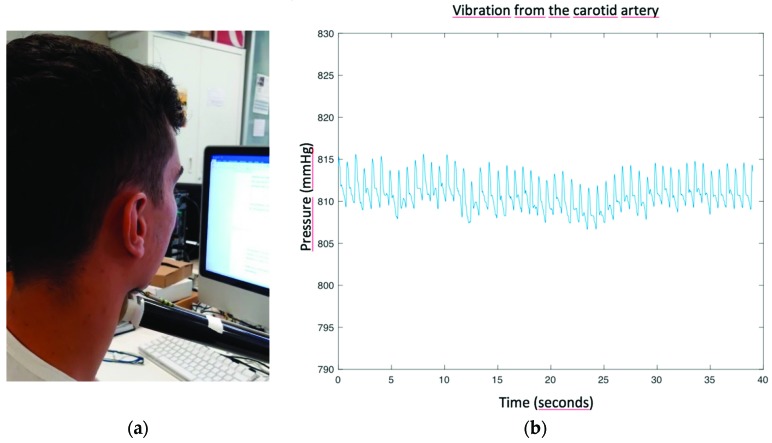
(**a**) Setup for carrying out pressure wave measurements; (**b**) stability in the pressure wave.

Before the measurement, the support is adjusted to the proper position for each user, and then it is fixed. To carry out the measurements, the user approaches the device until the device touches the user’s neck, as shown in Figure 2a. The combination of the fixed support and the buzzer system reduces the pressure fluctuations and achieves greater stability in the detection of the vibrations from the carotid artery, as shown in Figure 2b. In this picture, the plot of the pressure signal obtained from the vibrations recorded when a user was still and making the measurement is shown. It can be seen how the pressure remains within the targeted range (810.06–817.56 mmHg) throughout the whole measurement time. Consequently, this is translated into an improved signal quality by distinguishing the different key points in the blood pressure wave.

### 2.2. Validation of the Device

To assess the performance of the device, in this study, several sets of measurements were carried out with five healthy volunteers aged between 24 and 28 (Table 1 shows more information about the subjects). Each one of the users made five measurements lasting five minutes each, having five spare minutes between measurements. At the time of processing the data, they were processed independently to observe if there were significant changes.

**Figure 3:**
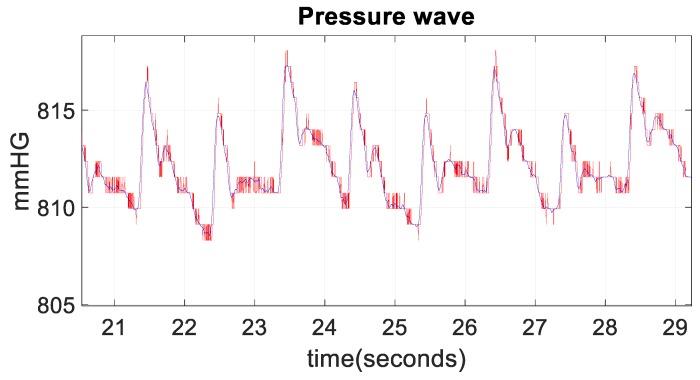
Raw pressure wave in red and smoothed and filtered wave in blue.

After each measurement, once the pressure values were obtained using the Arduino microcontroller, these values were loaded into MATLAB environment running in a computer. In order to improve the obtained wave quality and eliminate the noise, the pressure signal was smoothed and filtered. In order to do this, a low-pass filter with a cut-off frequency of 10 Hz was digitally applied. Figure 3 shows the raw pressure wave in red and the pressure wave in blue after the smoothing and filtering.

The blood pressure wave obtained has several key points corresponding to the phases of the cardiac cycle (systole and diastole). Systole is the contraction phase of the heart, where blood is pumped into the blood vessels, and diastole is the relaxation phase, which allows blood to enter the heart. The maximum pressure is obtained during the period of ventricular ejection, that is, in the systolic phase, whereas the minimum pressure is obtained at the final stage of diastole, prior to ventricular contraction. The dicrotic notch is the point that discriminates the passage from systole to diastole; this point corresponds to a transient increase in aortic pressure when the aortic valve is closed [23]. 

**Figure 4:**
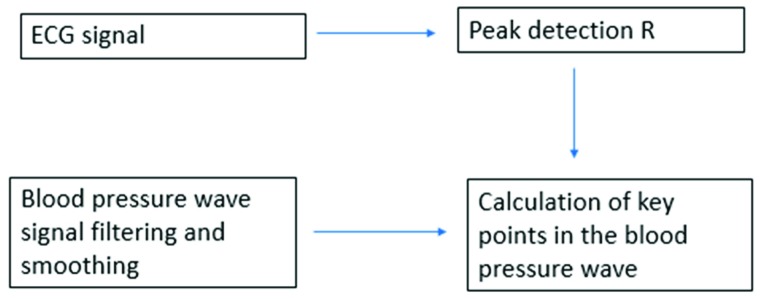
Algorithm used for the detection of key points of the blood pressure wave. ECG, electrocardiogram.

**Figure 5:**
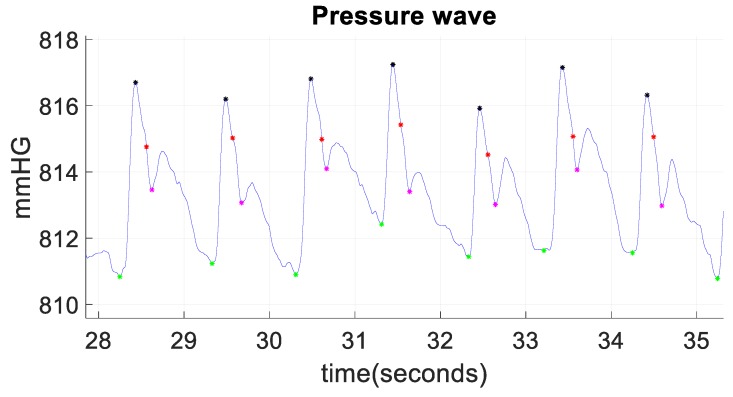
Key points of the blood pressure wave. Diastolic points in green, systolic points in black, dicrotic notches in magenta, and decay rate (30% pressure drops) in red.

Given the importance of these points when analyzing a blood pressure wave, an algorithm was performed to identify diastolic points (pressure signal minima), systolic points (pressure signal maxima), and dicrotic notches (transient increase in aortic pressure). This algorithm, depicted in Figure 4, is based on the calculation of the so-called R points of the ECG wave (systolic points corresponding to the contraction phase of the heart) and, from these, the maximum pressure points of the blood pressure wave can be obtained, knowing that they will happen moments later. Once the maximum in the pressure wave has been identified, it is easy to obtain the other characteristic points, as they will happen milliseconds later. Figure 5 shows the diastolic points in green, the systolic points in black, and the dicrotic notches in magenta. Moreover, by identifying the points with the highest and lowest pressure, the difference in pressure between the systolic point and the next diastolic point can be easily calculated. With this calculation, it is possible to analyze the decay rate (relaxation time) of the pressure wave, that is, to identify the value and the time in which the pressure drops a certain percentage (adjustable) with respect to the maximum pressure. This parameter allows to see if the blood pressure curve always has the same slope, and to what extent it varies. Figure 5 shows these points in red and, in this case, they correspond to a drop of 30% with respect to the systolic pressure.

**Figure 6:**
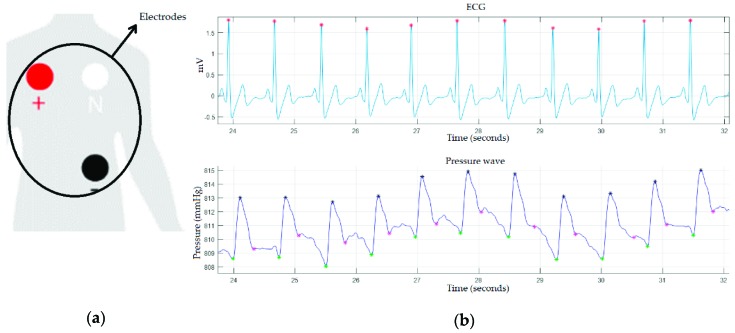
(**a**) Electrode arrangement; (**b**) ECG signal acquired through e-Health (top) and pressure wave (bottom).

As discussed above, the proposed device is capable of obtaining the ECG signal by means of the e-Health module, with the help of three electrodes (positive, negative, and neutral) attached to the body and arranged as shown in Figure 6a. The objective of obtaining the ECG signal is to monitor the pulse transit time, as well as the heart pulse variability. For this reason, R peaks (heart contraction phase) of the ECG signal were computed. In addition, ECG signal acquisition also helps in discerning key points, as mentioned above. The top of Figure 6b shows the ECG signal in blue and the R points corresponding to systole in red, while the pressure wave is plotted at the bottom of the figure.

**Figure 7:**
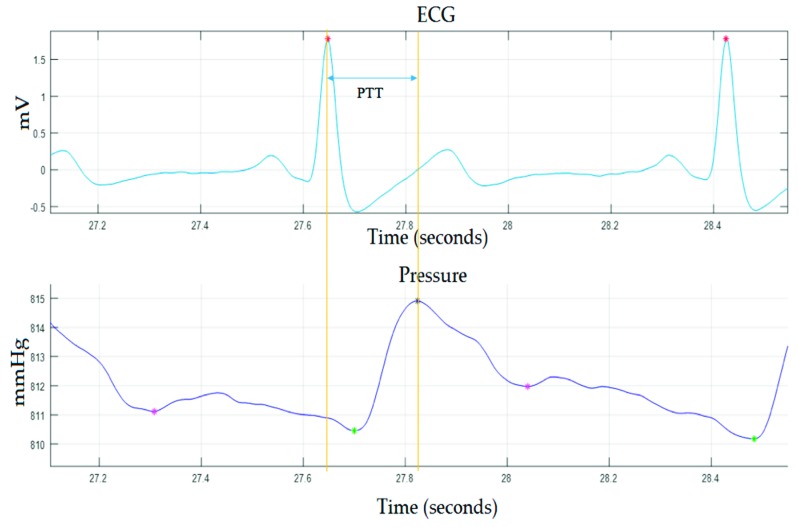
Pulse transit time (PTT).

Once the R peaks of the ECG signal are detected, it is easy to obtain the variability of the cardiac pulse (HRV), as this parameter is defined as the variation in the interval from beat to beat. The R peaks are also required for monitoring the PTT, because this parameter is defined as the time interval of the propagation of the pulse wave between two arterial sites [24,25]. Effectively, in this work, the time it takes for the pressure wave to propagate from the heart to the carotid artery was measured. To do so, the time between the R peak of the ECG signal and the next maximum pressure point (systolic pressure) was computed, as shown in Figure 7. 

**Figure 8:**
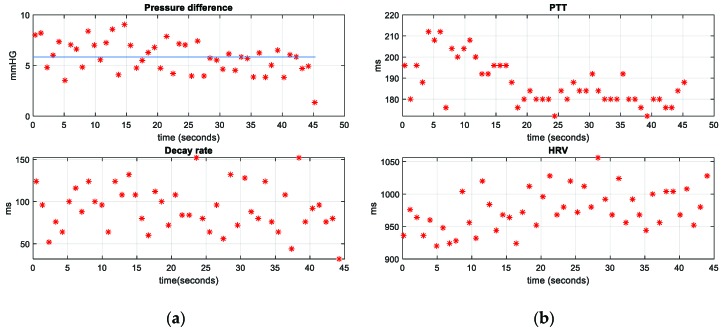
(**a**) Plots of pressure difference and decay rate against time; (**b**) plots of PTT and heart rate variability (HRV) against time.

When analyzing all the obtained data, this study has put the main focus on the pressure difference, decay rate, PTT, and HRV, plotted in Figure 8, for one of the volunteers making the measurements. In order to identify the average value of the pressure difference, the median was calculated (the blue line in the pressure difference plot in Figure 8a). This parameter was preferred instead of the mean owing to the noticeable influence of the extreme values on the mean. Thus, when the data do not show a normal distribution, the median provides a more robust estimator for the average value, which is relatively insensitive to the extreme values, making it more convenient in this case. As can be seen in Figure 8a, the values obtained from the pressure difference are close to the median, which means that the pressure difference does not vary excessively (∼5 mmHg). As far as the decay time is concerned, it can be observed how more dispersed data are obtained, which means that the pressure wave curve does not always have the same falling slope. In the graphs in Figure 8b, it can be seen how the PTT values drop from 210 to 180 (change of 14%), which means a small change in the blood pressure. As for the HRV parameter, it does not change abruptly, which indicates absence of arrhythmias. These results are logical given that the volunteers were healthy and had no detected disorders.

## 3. Results

**Figure 9:**
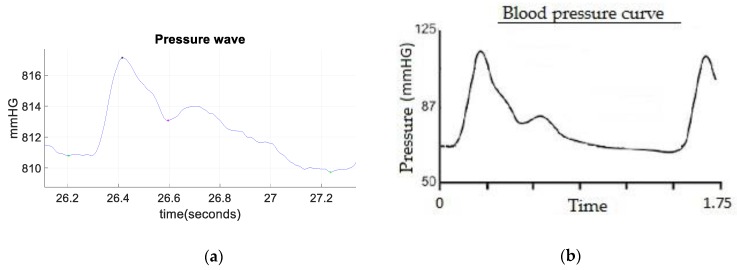
(**a**) Non-invasive blood pressure wave morphology; (**b**) invasive blood pressure wave morphology.

As shown throughout the paper, the pressure wave in the carotid artery can be successfully obtained non-invasively with the proposed device. The reliability of the device is acceptable, as can be inferred from Figure 9, as the shape of the obtained pressure wave is similar to the one obtained with a conventional invasive procedure. The morphology of the obtained signal can be seen in Figure 9a, which was taken in one of the tests performed with the volunteers. In it, the key points previously discussed can be discerned. With this wave, a rapid screening of the circulatory system can be made. This is especially useful in detecting the characteristic changes in the morphology of the arterial pressure wave owing to the arterial stiffness increase, which can be related to age as well as to numerous arterial alterations [26,27]. Compared with the blood pressure signal obtained in an invasive manner (using an intra-arterial catheter connected to a pressure transducer), shown in Figure 9b, the similarity of these waves is clear. The only difference between the two waves is that with the intraarterial pressure wave, the systolic and diastolic (maximum and minimum) blood pressure can be precisely known in a direct manner, making it easily monitorable in a clinical setting, whereas the system shown in this paper gives an indirect estimation of it. The main advantages of the proposed device are the reduced size, the cuffless condition, and the potential to be a continuous ambulatory device. Regarding the repeatability of the device, the narrow pressure range accepted (108 to 109 kPa) for the placement of the device and the buzzer with the aim of making sure this range is always met, allow to obtain a good repeatability, as the same conditions are ensured for all the measurements. This can be seen, for instance, in Figure 8a, given the low variations of the pressure differences obtained (∼5 mmHg).

With this device, it is possible to obtain other cardiac system monitoring parameters such as heart rate variability and pulse transit time. As the pressure wave can be acquired by means of the proposed device, it is expected to be able to estimate blood pressure values with a previous calibration with a sphygmomanometer. Provided that both the placement and the force exerted against the sensor during the measurements are kept constant, the estimation of the values of the arterial pressure can be achieved.

In Table 2, a comparison between the proposed device in this work and the system proposed in the works of [17,28] is shown. This table gathers the most interesting characteristics of this device and compares them to the achievements from different state-of-the-art approaches by other authors. As can be seen, this proposal is not able to provide for blood pressure estimation (although it could do it indirectly, as discussed), but it does obtain HRV and provides for pressure waveform analysis. In addition, albeit the current implementation is not wearable, it is a relatively small device, and a wearable version could be easily developed in the future with a system to stably attach the device to the user’s neck.

## 4. Discussion

Once the present device has been described, the results will be evaluated and compared with relevant studies. The device proposed in the work of [17] is wearable and noninvasive (PTT), it is the size of a bracelet, and it is capable of estimating blood pressure but not heart rate variability. The device proposed in the work of [28] shows some similarities with the one proposed in this work, although it has the advantage that it is totally wearable. In another paper [29], SphygmoCor Vx is proposed, which is a non-invasive system that measures the velocity of the pressure wave. This device has a great precision and capacity to perform the analysis of the pressure wave, although it is very bulky and not ambulatory. In Table 2, the advantages of the device in this work with respect to the above-mentioned devices are highlighted, with the ability to perform pressure waveform analysis being the most noticeable.

As has been shown, the device presented in this article is part of a more complex system that monitors biomedical variables (ECG, accelerometer, GSR) on an outpatient basis in patients suffering from epileptic seizures. The main advantages of the proposed device are the reduced size (compared with the works of [17,29]), the cuffless condition, and the potential to be a continuous ambulatory device. These features could be exploited in ambulatory tests.

With the obtention of the arterial pressure wave by means of the device, screening of the circulatory system can be made. It is thus expected that further progress will be made in this field and that the device described in this article will serve as a basis for monitoring and estimating blood pressure values (systolic and diastolic pressure) in a non-invasive way by correlating them to the parameters described in Figure 8. As future scope, the system presented in this work is expected to be tested with patients suffering from arrhythmias and changes in blood pressure, to assess its screening capabilities in a comprehensive manner. Improvements to the device are also foreseen, providing for wearable versions with even smaller sizes.

## Figures and Tables

**Table 1:** Information about the subjects.

Gender	Age (Years)	Weight (kg)
Female	28	65
Male	28	77
Male	27	82
Male	25	74
Male	24	76

**Table 2:** Comparison of the proposed device in this work and the proposed in the works of [17,28].

Characteristics	This Device	Device in [17]	Device in [28]
Wearable	Currently not	Yes	Yes
Non-invasive	Yes	Yes	Yes
Small size	Yes	No	Yes
Blood pressure estimation	No	Yes	No
Stability	Yes	Yes	Yes
HRV	Yes	No	Yes
Pressure waveform analysis	Yes	No	No
